# The angiotensin II/AT1 receptor pathway mediates malaria-induced acute kidney injury

**DOI:** 10.1371/journal.pone.0203836

**Published:** 2018-09-11

**Authors:** Leandro S. Silva, Diogo B. Peruchetti, Rodrigo P. Silva-Aguiar, Thiago P. Abreu, Beatriz K. A. Dal-Cheri, Christina M. Takiya, Mariana C. Souza, Maria G. Henriques, Ana Acacia S. Pinheiro, Celso Caruso-Neves

**Affiliations:** 1 Instituto de Biofísica Carlos Chagas Filho, Universidade Federal do Rio de Janeiro, Rio de Janeiro, RJ, Brazil; 2 Instituto de tecnologia em Fármacos, Fundação Oswaldo Cruz, Rio de Janeiro, RJ, Brazil; 3 Instituto Nacional de Ciência e Tecnologia em Medicina Regenerativa, Conselho Nacional de Desenvolvimento Científico e Tecnológico/MCT, Rio de Janeiro, RJ, Brazil; Max Delbruck Centrum fur Molekulare Medizin Berlin Buch, GERMANY

## Abstract

Malaria-induced acute kidney injury (MAKI) is a life-threatening complication of severe malaria. Here, we investigated the potential role of the angiotensin II (Ang II)/AT_1_ receptor pathway in the development of MAKI. We used C57BL/6 mice infected by *Plasmodium berghei* ANKA (PbA-infected mice), a well-known murine model of severe malaria. The animals were treated with 20 mg/kg/day losartan, an antagonist of AT_1_ receptor, or captopril, an angiotensin-converting enzyme inhibitor. We observed an increase in the levels of plasma creatinine and blood urea nitrogen associated with a significant decrease in creatinine clearance, a marker of glomerular flow rate, and glomerular hypercellularity, indicating glomerular injury. PbA-infected mice also presented proteinuria and a high level of urinary γ-glutamyltransferase activity associated with an increase in collagen deposition and interstitial space, showing tubule-interstitial injury. PbA-infected mice were also found to have increased fractional excretion of sodium (FE_Na+_) coupled with decreased cortical (Na^+^+K^+^)ATPase activity. These injuries were associated with an increase in pro-inflammatory cytokines, such as tumor necrosis factor alpha, interleukin-6, interleukin-17, and interferon gamma, in the renal cortex of PbA-infected mice. All modifications of these structural, biochemical, and functional parameters observed in PbA-infected mice were avoided with simultaneous treatment with losartan or captopril. Our data allow us to postulate that the Ang II/AT_1_ receptor pathway mediates an increase in renal pro-inflammatory cytokines, which in turn leads to the glomerular and tubular injuries observed in MAKI.

## Introduction

Malaria is one of the main causes of death from infectious disease worldwide [1]. *Plasmodium falciparum* infection induces the most severe form of malaria, leading to life-threatening complications such as cerebral malaria (CM), lung injury and acute kidney injury (AKI) [2–6]. Renal disease is correlated with high mortality in patients with malaria [2,7–9]. Remarkably, there is a strict correlation between the renin-angiotensin system (RAS) and the severity of malaria [10,11]. In severe malaria, activation of the sympathetic nervous system has been observed, due to vasodilation, which in turn leads to stimulation of RAS and a consequent increase in the level of angiotensin II (Ang II) [9].

The effects of Ang II are mediated by specific receptors: AT_1_ and AT_2_ [12–14]. The Ang II/AT_1_ receptor pathway plays a central role in the development of the glomerular and tubular injuries observed in AKI from different causes. This effect has been associated with the induction of a pro-inflammatory phenotype promoting immune cell infiltration and cytokine secretion in renal tissue [15–18]. It has been shown that pro-inflammatory cytokine production is strongly associated with severe malaria [19,20]. Previously, the Ang II/AT_1_ receptor pathway was implicated in the modulation of immune cells such as CD4^+^ and CD8^+^ T cells and brain damage in experimental CM, modulating the secretion of pro-inflammatory cytokines [10,11,21–23].

Therefore, it is possible to postulate that the Ang II/AT_1_ receptor pathway is involved in the development of MAKI. To test this hypothesis, in this work we used a well-known murine model of severe malaria, C57BL/6 mice infected by *P*. *berghei* ANKA (PbA-infected mice) [10,24–26], treated or not with losartan or captopril, blockers of the Ang II/AT_1_ receptor pathway. We observed that these compounds abolished the increase in secretion of pro-inflammatory cytokines, such as interferon gamma (IFN-γ), interleukin (IL)-6, tumor necrosis factor alpha (TNF-α), and IL-17, avoiding the development of glomerular and tubular injuries in MAKI. These data help us to better clarify the molecular mechanism of pathogenesis of MAKI and suggest a potential strategy for adjuvant treatment with RAS inhibitors in human malaria.

## Materials and methods

### Animals and experimental protocol

C57BL/6 male mice (6–8 weeks old) were obtained from the Institute of Science and Technology in Biomodels (ICTB) of the Oswaldo Cruz Foundation (FIOCRUZ), Rio de Janeiro, Brazil. The animals were accommodated in an air-conditioned environment (22–24°C) in a regular 12-h light/dark cycle with water and standard feed *ad libitum*.

Mice were randomly sorted into four groups: (1) non-infected mice (control group); (2) *P*. *berghei*/ANKA (PbA)-infected mice (vehicle group); (3) PbA-infected mice treated with losartan (los group); and (4) PbA-infected mice treated with captopril (cap group). The animal were infected by intraperitoneal injection of 10^6^ infected red blood cells with PbA obtained from mice of the same background, as described previously [10,25]. Peripheral blood parasitemia was determined using bright-field microscopy by a blind counter in a thick blood smear stained with Diff-Quick. When indicated, the animals were treated with 20 mg/kg/day losartan or captopril via gavage for 5 consecutive days from the day of PbA infection.

All procedures involving the handling of animals were carried out in accordance with the Guide for the Care and Use of Laboratory Animals of the National Institutes of Health. The experimental protocol was previously submitted to the Institutional Ethics Committee of Federal University of Rio de Janeiro and approved under permit number 008/2018. During the course of the study, the presence or absence of adverse clinical signs associated with C57BL6 strain such as hydrocephalus, microphthalmia, anopthalmia, malocclusion, barbering and ulcerative dermatitis were checked out. In addition, other possible abnormalities such as skin lesions, occurrence of tumors, problem of the eye, hydration status, body condition, and abnormalities in the teeth, genitals and abdomen were analyzed. Furthermore, general behavior aspects such as mobility degree inside cage, interaction with cage mates, eating, drinking, absence of feces or diarrhea and the capability of the animals to build a nest were also monitored. In order to minimize suffering, the animals were euthanized with a combination of the following anesthetics: ketamine (240 mg/kg body weight) and xylazine (15 mg/kg body weight). The kidneys and blood were then collected for analysis.

### Renal function analysis

To determine renal function, the volume of urine accumulated for 24 h (at day 5 post infection) was measured and the urinary flow calculated. In addition, a urine sample was collected, clarified by centrifugation (600 × *g* for 5 min), and the supernatant used to analyze creatinine, sodium excretion, urinary γ-glutamyltransferase (GT) activity, and proteinuria. As reported previously [25,27–33], to reduce physiologic changes induced by a change in environment, the mice were kept in metabolic cages for 24 h before sample collection. Plasma samples were also obtained to analyze creatinine, sodium, and blood urea nitrogen levels. The creatinine levels were determined by the alkaline picrate method (Gold Analisa Kit #335, Belo Horizonte, MG, Brazil). The levels of urinary protein were determined by the pyragallol red method (Labtest Kit #36, Lagoa Santa, MG, Brazil) or by the in-gel protein detection method using Coomassie dye staining. Urinary γ-GT activity was determined by its enzyme activity (Bioclin Kit #K080, Belo Horizonte, MG, Brazil). Sodium levels were analyzed by the photometric colorimetric test (Human Diagnostics Kit #573351, Wiesbaden, Germany). Creatinine clearance (CCr), urinary protein/urinary creatinine (UP:Cr) ratio, and fractional excretion of sodium (FE_Na+_) were calculated.

### Histology and histomorphometric studies

Before kidney extraction, the euthanized mice were perfused with saline and 4% paraformaldehyde using a peristaltic pump with a flow rate of 10 mL/min. The kidneys were then removed, segmented in midsagittal into two parts, which were maintained in Gendre fixative solution for 24 h. Next, the kidneys were fixed for 48 h in 10% buffered formalin and subsequently impregnated in paraffin. Histologic sections (4-μm thick) of kidney were obtained and stained with periodic acid-Schiff reagent (Sigma-Aldrich, St Louis, MA) for analysis of the glomerular cellularity and the area of tubule-interstitial space.

To assess tissue collagen deposition, 7-μm-thick sections were prepared and stained with Picrosirius red stain (Sigma-Aldrich, St. Louis, MA). Images were obtained using a Nikon 80i eclipse microscope (Nikon, Japan) and the analysis and quantification were performed using Image-Pro Plus image analysis software (Media Cybernetics, Inc., USA) in at least 15 randomly captured photomicrographs [24,27–30].

### (Na^+^+K^+^)ATPase activity assay in renal cortex homogenate

The kidneys were removed and homogenized in a cold solution containing 10 mM HEPES-Tris (pH 7.6), 250 mM sucrose, 2 mM EDTA, and 1 mM phenylmethylsulfonyl fluoride. Homogenates were centrifuged at 7000 *× g* at 4°C for 10 min, and the final supernatant was stored at –80°C [27,28,30]. Total protein concentrations were determined by the Folin phenol method [31].

An ATPase activity assay was performed on the renal cortex homogenate fraction as described previously [32–34]. Briefly, the reaction medium was composed of 10 mmol/L MgCl_2_, 20 mmol/L HEPES-Tris (pH 7.0), 30 mmol/L KCl, 120 mmol/L NaCl, and 5 mmol/L ATP (specific activity 0.27 μCi/nmol [γ^32^P]ATP). [γ^32^P]ATP was used as a tracer. The reaction was started with the addition of homogenate samples at final protein concentrations ranging from 0.3 to 0.5 mg/mL. After 10 min at 37°C, the reaction was stopped with cold charcoal activated by 0.1 N HCl. After centrifugation for 5 min at 1255 × *g*, the supernatant was harvested and the ^32^Pi released was measured by liquid scintillation counter (Packard Tri-Carb 2100 TR). The specific (Na^+^+K^+^)ATPase activity was assessed from the mathematical difference between released [^32^P]Pi values in the absence and in the presence of 1 mM ouabain (a specific inhibitor of (Na^+^+K^+^)ATPase).

### Cytokines

Cytokines levels in the renal cortex were determined as described earlier [24,27,29]. Briefly, TNF-α, IL-6, and IL-17 concentrations in renal cortex homogenate were evaluated by cytometric bead array (BD Biosciences, San Jose, CA), according to the manufacturer’s instructions. The results were expressed as ng/μg of protein.

### Statistical analysis

Statistical significance was assessed using ANOVA followed by multiple comparative Newman-Keuls test. GraphPad Prism version 5 (GraphPad Software, San Diego, CA) was used for analysis. The results are expressed as the means ± standard error of 2 representative experiments. Each experiment was carried out using 4 or 5 animals per group with the exception of the cytokines and BUN analyses (2 or 3 animals per group), and the differences were considered significant when *P* < 0.05.

## Results

### Role of the Ang II/AT_1_ receptor pathway in glomerular and tubular injuries in PbA-infected mice

**Fig 1:**
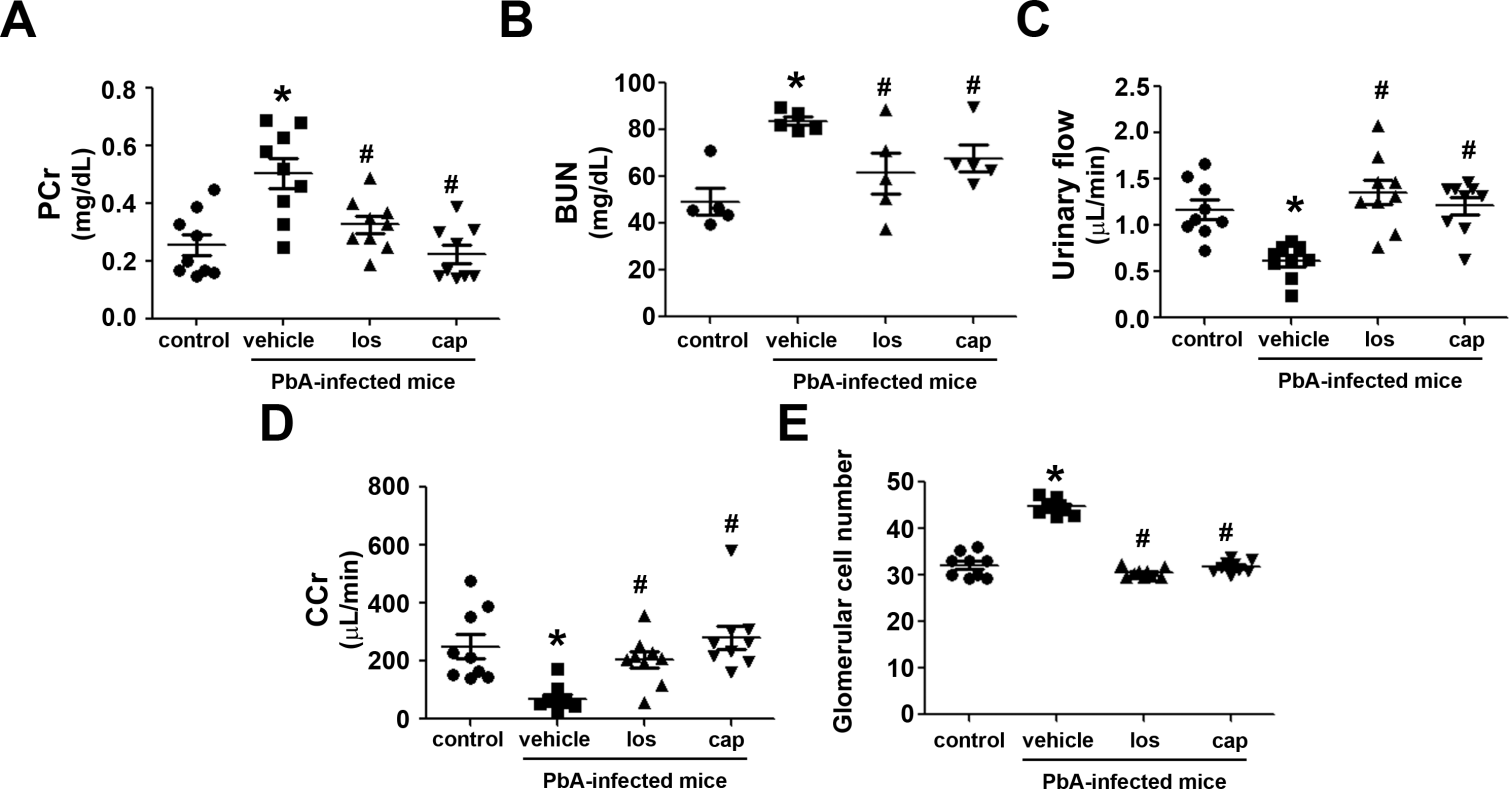
Ang II/AT_1_ receptor pathway mediates glomerular injury in PbA-infected mice. Male C57BL/6 mice were infected with *P*. *berghei*/ANKA (PbA-infected mice) and then, when indicated, simultaneously treated with 20 mg/kg/day of losartan or captopril over 5 days as described in the Materials and methods section (n = 9 per group except the blood urea nitrogen analysis, n = 5). **A**) Plasma creatinine. **B**) Blood urea nitrogen. **C**) Urinary flow. **D**) Creatinine clearance. **E**) Quantification of glomerular cellularity. PCr, plasma creatinine; BUN, blood urea nitrogen; CCr, creatinine clearance. The results are expressed as means ± SE. Statistically significant in relation to control (*P < 0.05) and vehicle (^#^*P* < 0.05).

Treatment of PbA-infected mice with losartan and captopril was done simultaneously with infection, which allowed us to study the role of the Ang II/AT_1_ receptor pathway on the development of MAKI. Initially, we measured renal parameters correlated to glomerular functions. PbA-infected mice presented an increase in plasma creatinine and blood urea nitrogen (Fig 1A and 1B), which is in agreement with the decrease in urinary flow and creatinine clearance (CCr), a marker of glomerular flow rate (Fig 1C and 1D). These results are in accordance with the increase in glomerular cellularity observed in PbA-infected mice (Fig 1E). Treatment with losartan or captopril avoided the development of glomerular injury in PbA-infected mice.

**Fig 2:**
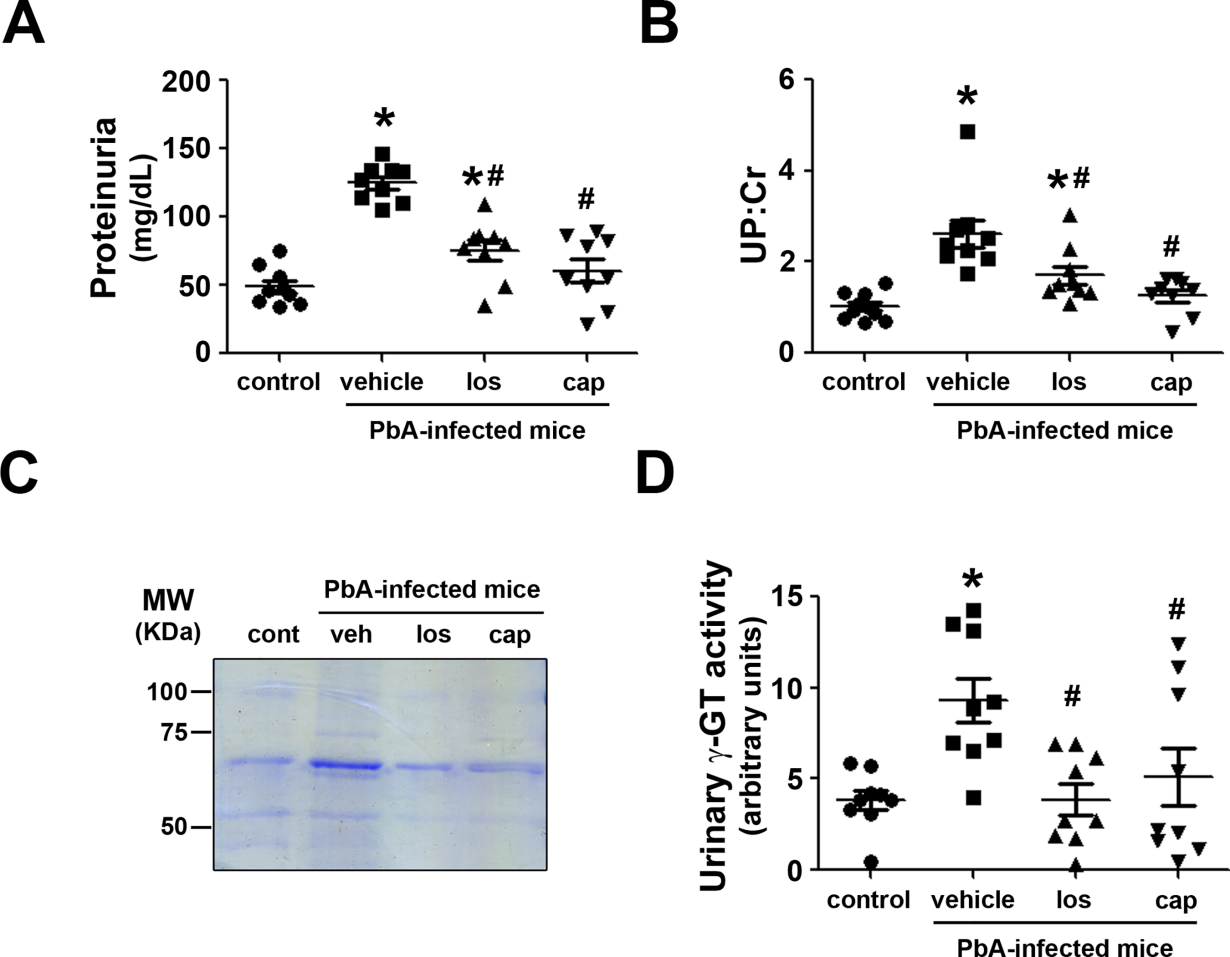
Ang II/AT_1_ receptor pathway mediates renal injury biomarkers in PbA-infected mice. Experimental groups are described in Fig 1 (n = 9 per group). **A**) Proteinuria. **B**) UP:Cr. **C**) Urinary protein profile. Urine samples were resolved on SDS-PAGE gels, and protein analysis was based on the intensity of Coomassie blue staining. **D**) Urinary γ-GT activity. UP:Cr, urinary protein/urinary creatinine ratio; γ-GT, γ-glutamyltransferase. The results are expressed as means ± SE. Statistically significant in relation to control (*P < 0.05) and vehicle (^#^P < 0.05).

**Fig 3:**
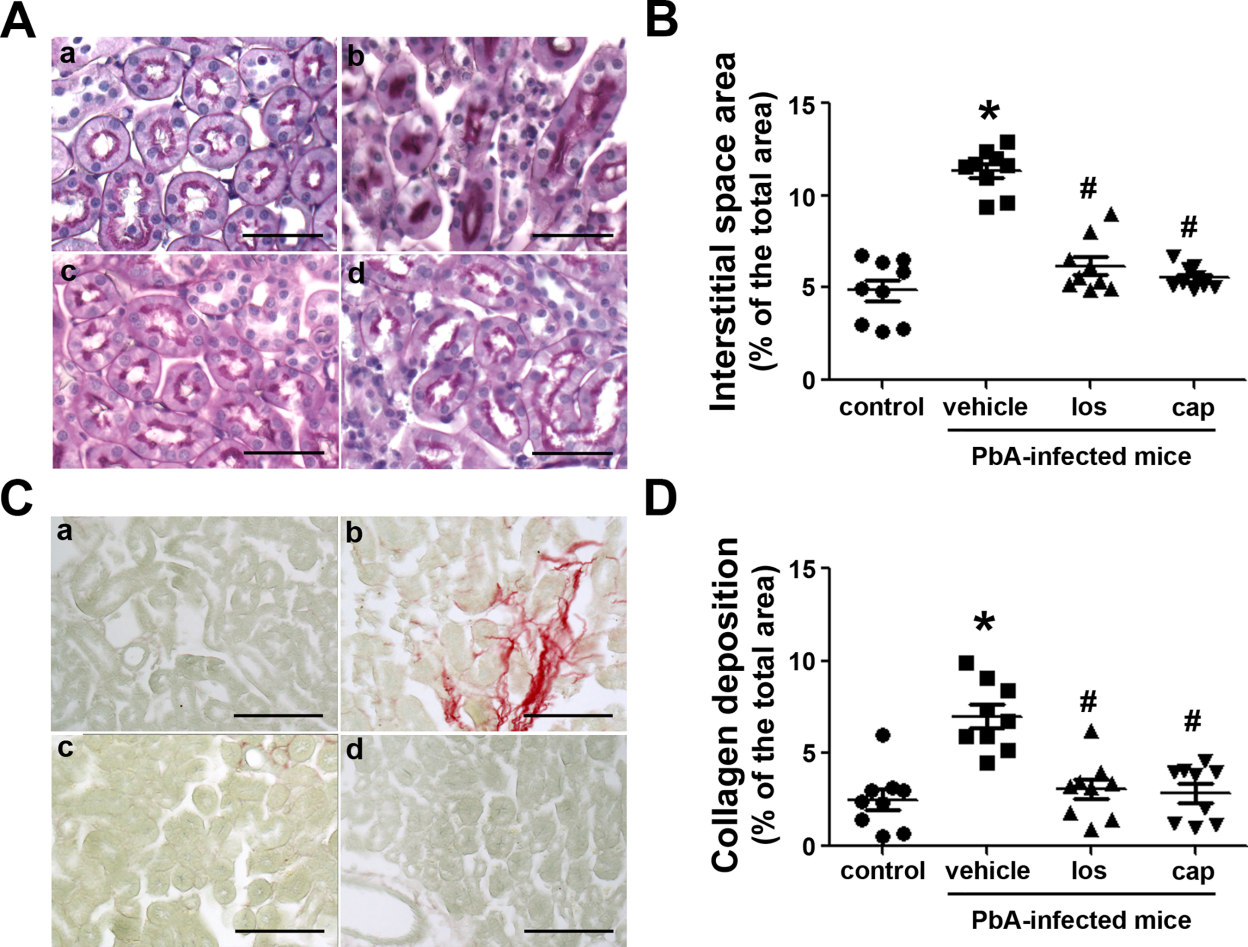
Ang II/AT_1_ receptor pathway mediates tubule-interstitial injury and fibrosis in PbA-infected mice. Experimental groups are described in Fig 1 (n = 9 per group). **A** and **B**) Periodic acid-Schiff stain was used to analyze the area of interstitial space in the renal cortex. **C** and **D**) Picrosirius red staining was used to determine collagen deposition in the renal cortex. a, uninfected and untreated mice control; b, PbA-infected mice; c, PbA-infected mice treated with 20 mg/kg/day of losartan; d, PbA-infected mice treated with 20 mg/kg/day of captopril. Scale bar, 50 μm. The results are expressed as means ± SE. Statistically significant in relation to control (*P < 0.05) and vehicle (^#^*P* < 0.05).

Proteinuria, a well-known marker of renal injury, was also evaluated (Fig 2). PbA-infected mice presented proteinuria as well as an increase in the urinary protein/creatinine (UP:Cr) ratio (Fig 2A–2C). Furthermore, the levels of urinary γ-GT, a marker of tubular injury, were also higher in PbA-infected mice (Fig 2D). In agreement with the renal injury markers, tubule-interstitial space and collagen deposition levels were increased in PbA-infected mice, indicating the development of a tubule-interstitial injury (Fig 3A–3D). All these parameters were ameliorated by treatment with losartan or captopril.

**Fig 4:**
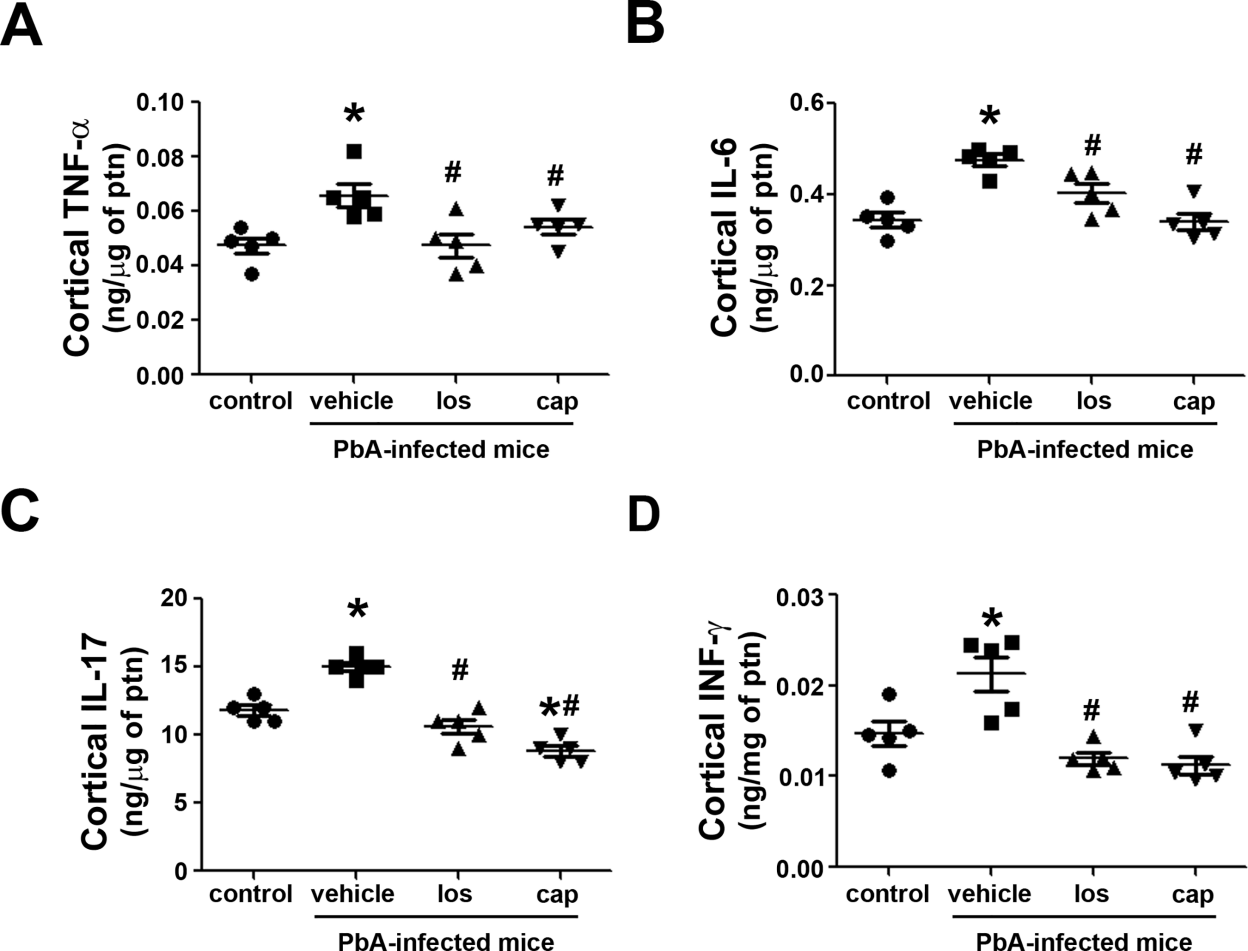
Ang II/AT_1_ receptor pathway mediates the increase in renal pro-inflammatory cytokines in PbA-infected mice. Experimental groups are described in Fig 1 (n = 5 per group). **A**) Cortical TNF-α, **B**) IL-6, **C**) IL-17, and **D**) IFN-γ levels were determined by ELISA. The cytokine levels were normalized by the amount of total protein in the same samples. TNF-α, tumor necrosis factor alpha; IL, interleukin; INF-γ, interferon gamma. The results are expressed as means ± SE. Statistically significant in relation to control (*P < 0.05) and vehicle (^#^*P* < 0.05).

AKI is characterized by an increase in pro-inflammatory cytokines in renal cortex segments [29,35–37]. Here, we measured the levels of TNF-α, IL-6, IL-17, and IFN-γ. The kidneys were perfused before the cortical region was isolated, avoiding serum cytokine contamination. The level of these pro-inflammatory cytokines was significantly increased in PbA-infected mice (Fig 4A–4D). The simultaneous treatment of with losartan or captopril blocked the secretion of these pro-inflammatory cytokines.

### Renal sodium handling in PbA-infected mice

**Fig 5:**
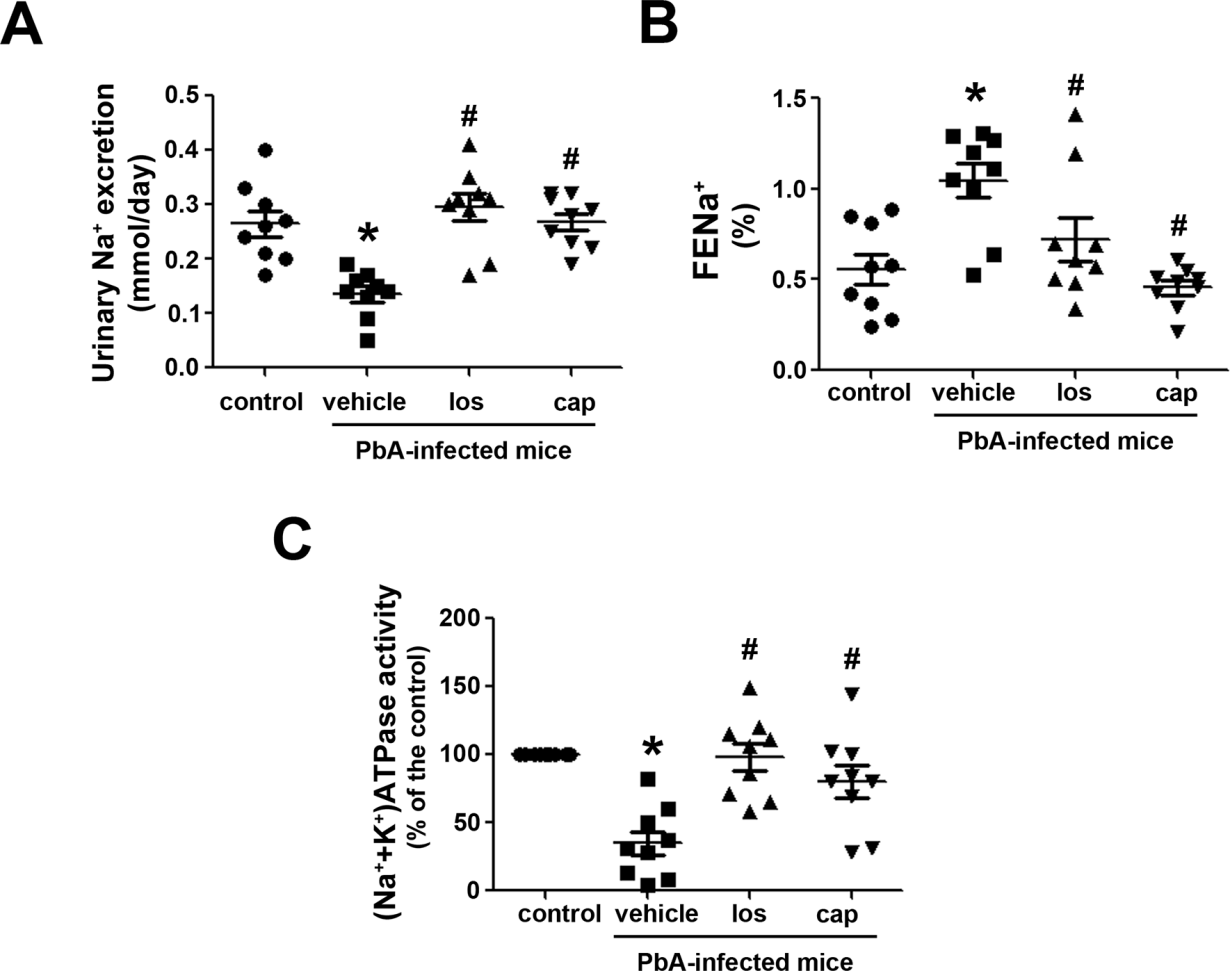
Ang II/AT_1_ receptor pathway mediates the changes of renal sodium handling in PbA-infected mice. Experimental groups are described in Fig 1 (n = 9 per group). **A**) Urinary Na^+^ excretion. **B**) FE_Na+_ levels. **C**) (Na^+^+K^+^)ATPase activity in the renal cortex. The results are expressed as means ± SE. Statistically significant in relation to control (*P < 0.05) and vehicle (^#^*P* < 0.05).

Another important characteristic of AKI is the change in renal sodium excretion [38,39]. Renal sodium handling depends on sodium reabsorption along the nephron, which is directly correlated to (Na^+^+K^+^)ATPase activity [33,34,40,41]. Here, it was observed that PbA-infected mice presented a decrease in urinary sodium excretion (Fig 5A). On the other hand, FE_Na+_, a marker of tubular sodium reabsorption, was increased in PbA-infected mice (Fig 5B). A decrease in (Na^+^+K^+^)ATPase activity was observed in the renal cortex of PbA-infected mice in accordance with the tubule-interstitial injury observed (Fig 5C). As observed for the other parameters, treatment with losartan or captopril completely avoided all changes in these parameters.

### Parasitemia and MAKI development in PbA-infected mice

It has been shown that there is a strict correlation between the development of MAKI and parasitemia [7–9]. One important question is whether the effect of Ang II/AT_1_ receptor pathway blockers could be due to a decrease in parasitemia. As observed in a previous study [10], peripheral blood parasitemia was also ameliorated by treatment with losartan or captopril (S1 Fig). However, significant parasitemia, about 10%, was still observed in PbA-infected mice treated with these compounds, indicating that a decrease in parasitemia is not directly responsible for the effect of losartan or captopril treatment. Together these data indicate that activation of the Ang II/AT_1_ receptor pathway is a critical step in the development of MAKI in severe malaria and probably involves a pro-inflammatory immune response.

## Discussion

MAKI is a life-threatening complication of severe malaria and is associated with higher mortality rates [2,7–9]. Thus, uncovering the mechanism underlying MAKI has become an important issue to understand the worsening prognosis of malaria. In the present work, we have shown the involvement of the renal Ang II/AT_1_ receptor pathway on both glomerular and tubular structural injuries in the development of MAKI, which involves modulation of the pro-inflammatory response. These data help to clarify the current understanding on the genesis of MAKI and will allow the development of new strategies for malaria co-adjuvant therapy.

Renal injury induced by malaria depends on the severity of the malaria infection [9]. PbA-infected mice are a well-known model of severe malaria associated with the development of AKI [24–26]. Here, PbA-infected mice showed a decrease in glomerular and tubular function with an increase in renal pro-inflammatory cytokines in agreement with previous studies [19,20,24–26,42]. In severe malaria, significant hypovolemia occurs, leading to activation of vasoactive mediators, which could be involved in the pathogenesis of malaria and associated diseases such as AKI [7–9,42]. Building on the idea that the Ang II/AT_1_ receptor pathway participates in the pathogenesis of malaria, we showed that this pathway is involved in the genesis of MAKI.

It has been proposed that the development of MAKI depends on parasite adhesion to renal endothelial cells as well as activation of the host immune response, which leads to glomerular and tubular injuries [7,8]. In a previous *in vitro* study, we showed that addition of Ang II decreases erythrocyte infection by *P*. *falciparum* through a mechanism that involves the production of Ang-(1–7) and, consequent activation of AT_1-7_ receptor [43].

In the present study, we observed that PbA-infected mice had 17% parasitemia at the 5^th^ day post infection. Parasitemia dropped to 10% when the animals were treated with losartan or captopril. Under these conditions, renal injury and secretion of pro-inflammatory cytokines were abolished. This result suggests that the decrease in parasitemia *per se* could be responsible for the lack of development of MAKI due to a delay in the immune response. However, Elias *et al*. [44], using a model of PbA-infected Balb/c mice, showed that even with low parasitemia (about 5%) the mice developed renal injury as well as the secretion of renal pro-inflammatory cytokines IL-6, TNF-α and INF-γ [44]. In agreement, Fu *et al*. [45], using both *Plasmodium yoelii* strain 17XNL (nonlethal) and 17XL (lethal), showed that despite different levels of peripheral parasitemia, both strains were able to enhance the release of peritoneal macrophage TNF-α and IL-6 from the first day post infection. Terkawai *et al*. [46], using C57BL/6 mice infected with *Plasmodium yoelii* 17XNL, observed multiorgan failure when the animals were depleted of phagocytic cells by treatment with clodronate, even with a decrease in parasitemia. Our results and those already published in the literature together indicate that MAKI development in malaria involves a more complex mechanism than modulation of the parasitemia level.

Here, we observed that losartan and captopril treatment abolished the renal pro-inflammatory response. In addition, it was shown that the Ang II/AT_1_ receptor pathway has a role in the establishment of an efficient T cell response in the spleen and therefore could participate in a misbalanced parasite-induced T cell immune response [10]. In murine malaria infection with *P*. *chabaudi* and *P*. *berghei*, an increase in intrarenal TNF-α and IL-6 levels was observed [47,48]. The involvement of these pro-inflammatory cytokines in renal tubule-interstitial injury has been demonstrated [27,29,35–37]. Correlating with this observation, Ang II was shown to induce an inflammatory response in renal epithelial cells in tubule-interstitial injury [10,49]. Based on these observations, we propose that an immune response could play an important role in the effect of the Ang II/AT_1_ receptor pathway in MAKI.

Our observations also suggest that changes in renal hemodynamics could contribute to the development of MAKI. It has been proposed that reduction in the glomerular filtration rate (GFR) in different forms of AKI is due to increased tubular fluid exacerbating the tubule-glomerular feedback mechanism [50]. This idea is strengthened by our observation that a decrease in tubular sodium reabsorption occurs, indicating an increase in distal fluid delivery causing a decrease in the GFR. On the other hand, the vasoconstrictor effect induced by activation of the Ang II/AT_1_ receptor pathway could be another component involved in the decrease in GFR observed in PbA-infected mice. Indeed, it is well known that Ang II promotes vasoconstriction preferentially of efferent arterioles, leading to a decrease in the renal plasma flow rate and a decrease in GFR [9,51].

Usually, tubule-interstitial injury observed in MAKI is associated with cytoadherence of infected erythrocytes to peritubular capillaries [7–9,42]. However, another interesting idea is the deleterious effect of protein overload in the lumen of proximal tubule (PT) [33,52–58]. It has been described that the Ang II/AT_1_ receptor pathway promotes an increase in glomerular permeability to albumin [59]. Some groups, including our group, have shown that a higher albumin concentration leads to secretion of pro-inflammatory mediators inducing tubule-interstitial injury [60–62]. In agreement, here we observed significant proteinuria in PbA-infected mice, indicating PT protein overload associated with an increase in pro-inflammatory cytokines. In addition, it has been proposed that protein overload in PT cells induces secretion of Ang II, which could mediate the deleterious effect of higher albumin concentration [63]. Thus, the positive feedback between glomerular and tubule-interstitial injuries could lead to a dangerous loop mediated by albumin and Ang II secretion. In fact, we observed an increase in proteinuria and in urinary γ-GT, a marker of PT cell injury. Interestingly, when the Ang II/AT_1_ receptor pathway was blocked, fibrosis, proteinuria, as well as the increase in the secretion of pro-inflammatory cytokines were abolished in PbA-infected mice, avoiding the development of tubule-interstitial injury observed in MAKI.

Together our data allow us to postulate that this increase in intrarenal pro-inflammatory cytokines observed in PbA-infected mice is mediated by the Ang II/AT_1_ receptor pathway, playing a critical role in the development of MAKI.

## Supporting information

S1 Fig Peripheral blood parasitemia on the 5th day post PbA infection. Experimental groups are described in Fig 1 (n = 9 per group). Peripheral blood parasitemia was determined in a blood smear stained with Diff-Quick. Scale bar, 20 μm. The results are expressed as means ± SE. Statistically significant in relation to vehicle (*P < 0.05).
(TIF)

## References

[1] WHO. World Malaria Report **2017**. Geneva: World Health Organization; 2017.

[2] Mishra SK, Mahanta KC, Mohanty S. Malaria associated acute renal failure experience from Rourkela, eastern India. J Indian Med Assoc. 2008; 106(10):640–2, 654. 19552096

[3] Prasad R, Mishra OP. Acute kidney injury in children with *Plasmodium falciparum* malaria: determinants for mortality. Perit Dial Int. 2016; 36(2):213–17. 10.3747/pdi.2014.00254 26429418 PMC4803368

[4] Boushab BM, Fall-Malick FZ, Savadogo M, Basco LK. Acute kidney injury in a shepherd with severe malaria: a case report. Int J Nephrol Renovasc Dis. 2016; 9:249–251. 27785088 10.2147/IJNRD.S116377 PMC5066854 27785088

[5] Taylor WR, Cañon V, White NJ. Pulmonary manifestations of malaria: recognition and management. Treat Respir Med. 2006; 5(6):419–28. 17154671 10.2165/00151829-200605060-00007

[6] Sercundes MK, Ortolan LS, Debone D, Soeiro-Pereira PV, Gomes E, Aitken EH, et al Targeting neutrophils to prevent malaria-associated acute lung injury/acute respiratory distress syndrome in mice. PLoS Pathog 2016; 12(12):e1006054 10.1371/journal.ppat.1006054 27926944 PMC5142790

[7] Krishnan A, Karnad DR. Severe falciparum malaria: an important cause of multiple organ failure in Indian intensive care unit patients. Crit Care Med. 2003; 31(9):2278–84. 10.1097/01.CCM.0000079603.82822.69 14501957

[8] Jones J, Holmen J, De Graauw J, Jovanovich A, Thornton S, Chonchol M. Association of complete recovery from acute kidney injury with incident CKD stage 3 and all-cause mortality. Am J Kidney Dis. 2012; 60(3):402–8. 10.1053/j.ajkd.2012.03.014 22541737 PMC3422603

[9] Sitprija V, Napathorn S, Laorpatanaskul S, Suithichaiyakul T, Moollaor P, Suwangool P, et al Renal and systemic hemodynamics in Falciparum malaria. Am J Nephrol. 1996; 16:513–19. 10.1159/000169042 8955763

[10] Silva-Filho JL, Souza MC, Ferreira-Dasilva CT, Silva LS, Costa MF, Padua TA, et al Angiotensin II is a new component involved in splenic T lymphocyte responses during *Plasmodium berghei* ANKA infection. PLoS One. 2013; 8(4):e62999 10.1371/journal.pone.0062999 23646169 PMC3639972

[11] Silva LS, Silva-Filho JL, Caruso-Neves C, Pinheiro AA. New concepts in malaria pathogenesis: the role of the renin-angiotensin system. Front Cell Infect Microbiol. 2016; 5:103 10.3389/fcimb.2015.00103 26779452 PMC4703750

[12] Fyhrquist F, Saijonmaa O. Renin-angiotensin system revisited. J Intern Med. 2008; 264(3):224–36. 10.1111/j.1365-2796.2008.01981.x 18793332 PMC7166930

[13] Lv LL, Liu BC. Role of non-classical renin-angiotensin system axis in renal fibrosis. Front Physiol. 2015; 6:117 10.3389/fphys.2015.00117 25954204 PMC4404823

[14] Carey RM. The intrarenal renin-angiotensin system in hypertension. Adv Chronic Kidney Dis. 2015; 22(3):204–10. 10.1053/j.ackd.2014.11.004 25908469

[15] Mehrotra P, Patel JB, Ivancic CM, Collett JA, Basile DP. Th-17 cell activation in response to high salt following acute kidney injury is associated with progressive fibrosis and attenuated by AT-1R antagonism. Kidney Int. 2015; 88(4):776–84. 10.1038/ki.2015.200 26200947 PMC4589446

[16] Rodríguez-Romo R, Benítez K, Barrera-Chimal J, Pérez-Villalva R, Gómez A, Aguilar-León D, et al AT1 receptor antagonism before ischemia prevents the transition of acute kidney injury to chronic kidney disease. Kidney Int. 2016; 89(2):363–73. 10.1038/ki.2015.320 26509589

[17] Zhang J, Rudemiller NP, Patel MB, Wei Q, Karlovich NS, Jeffs AD, et al Competing actions of type 1 angiotensin II receptors expressed on T lymphocytes and kidney epithelium during cisplatin-induced AKI. J Am Soc Nephrol. 2016; 27(8):2257–64. 10.1681/ASN.2015060683 26744488 PMC4978052

[18] Cheng SY, Chou YH, Liao FL, Lin CC, Chang FC, Liu CH, et al Losartan reduces ensuing chronic kidney disease and mortality after acute kidney injury. Sci Rep. 2016; 6:34265 10.1038/srep34265 27677327 PMC5039710

[19] Herbert F, Tchitchek N, Bansal D, Jacques J, Pathak S, Bécavin C, et al Evidence of IL-17, IP-10, and IL-10 involvement in multiple-organ dysfunction and IL-17 pathway in acute renal failure associated to *Plasmodium falciparum* malaria. J Transl Med. 2015; 13:369 10.1186/s12967-015-0731-6 26602091 PMC4658812

[20] Ding Y, Xu W, Zhou T, Liu T, Zheng H, Fu Y. Establishment of a murine model of cerebral malaria in KunMing mice infected with *Plasmodium berghei* ANKA. Parasitology. 2016; 143(12):1672–80. 10.1017/S0031182016001475 27574013

[21] Silva-Filho JL, Caruso-Neves C, Pinheiro AA. Targeting Angiotensin II Type-1 receptor (AT1R) inhibits the harmful phenotype of *Plasmodium*-specific CD8+ T cells during blood-stage malaria. Front Cell Infect Microbiol. 2017; 7:42 10.3389/fcimb.2017.00042 28261571 PMC5311040

[22] Silva-Filho JL, Caruso-Neves C, Pinheiro AA. Angiotensin II type-1 receptor (AT1R) regulates expansion, differentiation, and functional capacity of antigen-specific CD8+ T cells. Sci Rep. 2016; 6:35997 10.1038/srep35997 27782175 PMC5080615

[23] Gallego-Delgado J, Basu-Roy U, Ty M, Alique M, Fernandez-Arias C, Movila A, et al Angiotensin receptors and β-catenin regulate brain endothelial integrity in malaria. J Clin Invest. 2016; 126(10):4016–4029. 10.1172/JCI87306 27643439 PMC5096829

[24] Souza MC, Silva JD, Pádua TA, Torres ND, Antunes MA, et al Mesenchymal stromal cell therapy attenuated lung and kidney injury but not brain damage in experimental cerebral malaria. Stem Cell Res Ther. 2015; 6:102 10.1186/s13287-015-0093-2 25998168 PMC4462088

[25] Abreu TP, Silva LS, Takiya CM, Souza MC, Henriques MG, Pinheiro AA, et al Mice rescued from severe malaria are protected against renal injury during a second kidney insult. PLoS One. 2014; 9(4):e93634 10.1371/journal.pone.0093634 24736406 PMC3988045

[26] Pulido-Méndez M, Finol HJ, Girón ME, Aguilar I. Ultrastructural pathological changes in mice kidney caused by *Plasmodium berghei* infection. J Submicrosc Cytol Pathol. 2006; 38(2–3):143–8. 17784642

[27] Landgraf SS, Silva LS, Peruchetti DB, Sirtoli GM, Moraes-Santos F, Portella VG, et al 5-Lypoxygenase products are involved in renal tubulointerstitial injury induced by albumin overload in proximal tubules in mice. PLoS One. 2014; 9(10):e107549 10.1371/journal.pone.0107549 25302946 PMC4193734

[28] Landgraf SS, Wengert M, Silva JS, Zapata-Sudo G, Sudo RT, Takiya CM, et al Changes in angiotensin receptors expression play a pivotal role in the renal damage observed in spontaneously hypertensive rats. Am J Physiol Renal Physiol. 2011; 300(2):F499–510. 10.1152/ajprenal.00384.2010 21084406

[29] Portella VG, Silva-Filho JL, Landgraf SS, de Rico TB, Vieira MA, Takiya CM, et al Sepsis-surviving mice are more susceptible to a secondary kidney insult. Crit Care Med. 2013; 41(4):1056–68. 10.1097/CCM.0b013e3182746696 23385098

[30] Silva-Filho JL, Peruchetti DB, Moraes-Santos F, Landgraf SS, Silva LS, Sirtoli GM, et al Group V Secretory phospholipase A2 is involved in tubular integrity and sodium handling in the kidney. PLoS One. 2016; 11(1):e0147785 10.1371/journal.pone.0147785 26820468 PMC4731149

[31] Lowry OH, Rosebrough NJ, Farr AL, Randall RJ. Protein measurement with the Folin phenol reagent. J Biol Chem. 1951; 193(1):265–75. 14907713

[32] Queiroz-Madeira EP, Lara LS, Wengert M, Landgraf SS, Líbano-Soares JD, Zapata-Sudo G, et al Na(+)-ATPase in spontaneous hypertensive rats: possible AT(1) receptor target in the development of hypertension. Biochim Biophys Acta. 2010;1798(3):360–6. 10.1016/j.bbamem.2009.06.018 19560439

[33] Peruchetti DB, Pinheiro AA, Landgraf SS, Wengert M, Takiya CM, Guggino WB, et al (Na^+^ + K^+^)-ATPase is a target for phosphoinositide 3-kinase/protein kinase B and protein kinase C pathways triggered by albumin. J Biol Chem. 2011; 286(52):45041–7. 10.1074/jbc.M111.260737 22057272 PMC3247955

[34] Arnaud-Batista FJ, Peruchetti DB, Abreu TP, do Nascimento NR, Malnic G, Fonteles MC, et al Uroguanylin modulates (Na^+^+K^+^)ATPase in a proximal tubule cell line: Interactions among the cGMP/protein kinase G, cAMP/protein kinase A, and mTOR pathways. Biochim Biophys Acta. 2016; 1860(7):1431–8. 10.1016/j.bbagen.2016.04.012 27102282

[35] Ozkok A, Edelstein CL. Pathophysiology of cisplatin-induced acute kidney injury. Biomed Res Int. 2014; 2014:967826 10.1155/2014/967826 25165721 PMC4140112

[36] Malhotra R, Siew ED. Biomarkers for the early detection and prognosis of acute kidney injury. Clin J Am Soc Nephrol. 2017; 12(1):149–173. 10.2215/CJN.01300216 27827308 PMC5220647

[37] Bonavia A, Singbartl K. A review of the role of immune cells in acute kidney injury. Pediatr Nephrol. 2017 10.1007/s00467-017-3774-5 28801723

[38] Maciel AT, Vitorio D. Urine biochemistry assessment in critically ill patients: controversies and future perspectives. J Clin Monit Comput. 2017; 31(3):539–546. 10.1007/s10877-016-9871-3 27038161

[39] Vallon V. Tubular transport in acute kidney injury: relevance for diagnosis, prognosis and intervention. Nephron. 2016; 134(3):160–166. 10.1159/000446448 27238156 PMC5089910

[40] Féraille E, Doucet A. Sodium-potassium-adenosinetriphosphatase-dependent sodium transport in the kidney: hormonal control. Physiol Rev. 2001; 81(1):345–418. 10.1152/physrev.2001.81.1.345 11152761

[41] Taub M, Springate JE, Cutuli F. Targeting of renal proximal tubule Na,K-ATPase by salt-inducible kinase. Biochem Biophys Res Commun. 2010; 393(3):339–44. 10.1016/j.bbrc.2010.02.037 20152810 PMC2884295

[42] Eiam-Ong S, Sitprija V. Falciparum malaria and the kidney: a model of inflammation. Am J Kidney Dis. 1998; 32(3):361–75. 10.1053/ajkd.1998.v32.pm9740151 9740151

[43] Saraiva VB, de Souza Silva L, Ferreira-DaSilva CT, da Silva-Filho JL, Teixeira-Ferreira A, Perales J, et al Impairment of the *Plasmodium falciparum* erythrocytic cycle induced by angiotensin peptides. PLoS One. 2011; 6(2):e17174 10.1371/journal.pone.0017174 21364758 PMC3041794

[44] Elias RM, Correa-Costa M, Barreto CR, Silva RC, Hayashida CY, et al Oxidative stress and modification of renal vascular permeability are associated with acute kidney injury during *P*. *berghei* ANKA infection. PLoS One. 2012; 7(8):e44004 10.1371/journal.pone.0044004 22952850 PMC3432099

[45] Fu Y, Ding Y, Zhou TL, Ou QY, Xu WY. Comparative histopathology of mice infected with the 17XL and 17XNL strains of *Plasmodium yoelii*. J Parasitol. 2012; 98(2):310–5. 10.1645/GE-2825.1 22017443

[46] Terkawi MA, Nishimura M, Furuoka H, Nishikawa Y. Depletion of phagocytic cells during nonlethal *Plasmodium yoelii* infection causes severe malaria characterized by acute renal failure in mice. Infect Immun. 2016; 84(3):845–55. 10.1128/IAI.01005-15 26755155 PMC4771365

[47] Sinniah R, Rui-Mei L, Kara A. Up-regulation of cytokines in glomerulonephritis associated with murine malaria infection. Int J Exp Pathol. 1999; 80(2):87–95. 10.1046/j.1365-2613.1999.00101.x 10469263 PMC2517762

[48] Chang KH, Stevenson MM. Effect of anemia and renal cytokine production on erythropoietin production during blood-stage malaria. Kidney Int. 2004; 65(5):1640–6. 10.1111/j.1523-1755.2004.00573.x 15086902

[49] Norlander AE, Saleh MA, Kamat NV, Ko B, Gnecco J, Zhu L, et al Interleukin-17A regulates renal sodium transporters and renal injury in angiotensin II-Induced hypertension. Hypertension. 2016; 68(1):167–74. 10.1161/HYPERTENSIONAHA.116.07493 27141060 PMC4900947

[50] Matejovic M, Ince C, Chawla LS, Blantz R, Molitoris BA, Rosner MH, et al Renal hemodynamics in AKI: in search of new treatment targets. J Am Soc Nephrol. 2016; 27(1):49–58. 10.1681/ASN.2015030234 26510884 PMC4696587

[51] Navar LG, Gilmore JP, Joyner WL, Steinhausen M, Edwards RM, Casellas D, et al Direct assessment of renal microcirculatory dynamics. Fed Proc. 1986; 45(13):2851–61. 3780993

[52] Peruchetti DB, Cheng J, Caruso-Neves C, Guggino WB. Mis-regulation of mammalian target of rapamycin (mTOR) complexes induced by albuminuria in proximal tubules. J Biol Chem. 2014; 289(24):16790–801. 10.1074/jbc.M114.549717 24790108 PMC4059122

[53] Macconi D, Remuzzi G, Benigni A. Key fibrogenic mediators: old players. Renin-angiotensin system. Kidney Int Suppl (2011). 2014; 4(1):58–64. 10.1038/kisup.2014.11 26312151 PMC4536968

[54] Rüster C, Wolf G. Angiotensin II as a morphogenic cytokine stimulating renal fibrogenesis. J Am Soc Nephrol. 2011; 22(7):1189–99. 10.1681/ASN.2010040384 21719784

[55] Abbate M, Zoja C, Remuzzi G. How does proteinuria cause progressive renal damage? J Am Soc Nephrol. 2006; 17(11):2974–84. 10.1681/ASN.2006040377 17035611

[56] Caruso-Neves C, Pinheiro AA, Cai H, Souza-Menezes J, Guggino WB. PKB and megalin determine the survival or death of renal proximal tubule cells. Proc Natl Acad Sci U S A. 2006; 103(49):18810–5. 10.1073/pnas.0605029103 17121993 PMC1693744

[57] Gorriz JL, Martinez-Castelao A. Proteinuria: detection and role in native renal disease progression. Transplant Rev (Orlando). 2012; 26(1):3–13. 10.1016/j.trre.2011.10.002 22137726

[58] Erkan E. Proteinuria and progression of glomerular diseases. Pediatr Nephrol. 2013; 28(7):1049–58. 10.1007/s00467-012-2335-1 23124512

[59] Königshausen E, Zierhut UM, Ruetze M, Potthoff SA, Stegbauer J, Woznowski M, et al Angiotensin II increases glomerular permeability by β-arrestin mediated nephrin endocytosis. Sci Rep. 2016; 6:39513 10.1038/srep39513 28004760 PMC5177899

[60] Lv LL, Feng Y, Wen Y, Wu WJ, Ni HF, Li ZL, et al Exosomal CCL from tubular epithelial cells is critical for albumin-induced tubulointerstitial inflammation. J Am Soc Nephrol. 2018; 10.1681/ASN.2017050523 29295871 PMC5827595

[61] Liu D, Xu M, Ding LH, Lv LL, Liu H, Ma KL, et al Activation of the Nlrp3 inflammasome by mitochondrial reactive oxygen species: a novel mechanism of albumin-induced tubulointerstitial inflammation. Int J Biochem Cell Biol. 2014; 57:7–19. 10.1016/j.biocel.2014.09.018 25281528 PMC4414121

[62] Landgraf SS, Silva LS, Peruchetti DB, Sirtoli GM, Moraes-Santos F, Portella VG, et al 5-Lypoxygenase products are involved in renal tubulointerstitial injury induced by albumin overload in proximal tubules in mice. PLoS One. 2014; 9(10):e107549 10.1371/journal.pone.0107549 25302946 PMC4193734

[63] Wolf G, Schroeder R, Ziyadeh FN, Stahl RA. Albumin up-regulates the type II transforming growth factor-beta receptor in cultured proximal tubular cells. Kidney Int. 2004; 66(5):1849–58. 10.1111/j.1523-1755.2004.00958.x . 15496155

