# Supplementary figures and images for: The angiotensin II/AT1 receptor pathway mediates malaria-induced acute kidney injury
Source: PLoS One. 2018 Sep 11;13(9):e0203836. doi: 10.1371/journal.pone.0203836 (PMC6133374; doi:10.1371/journal.pone.0203836)

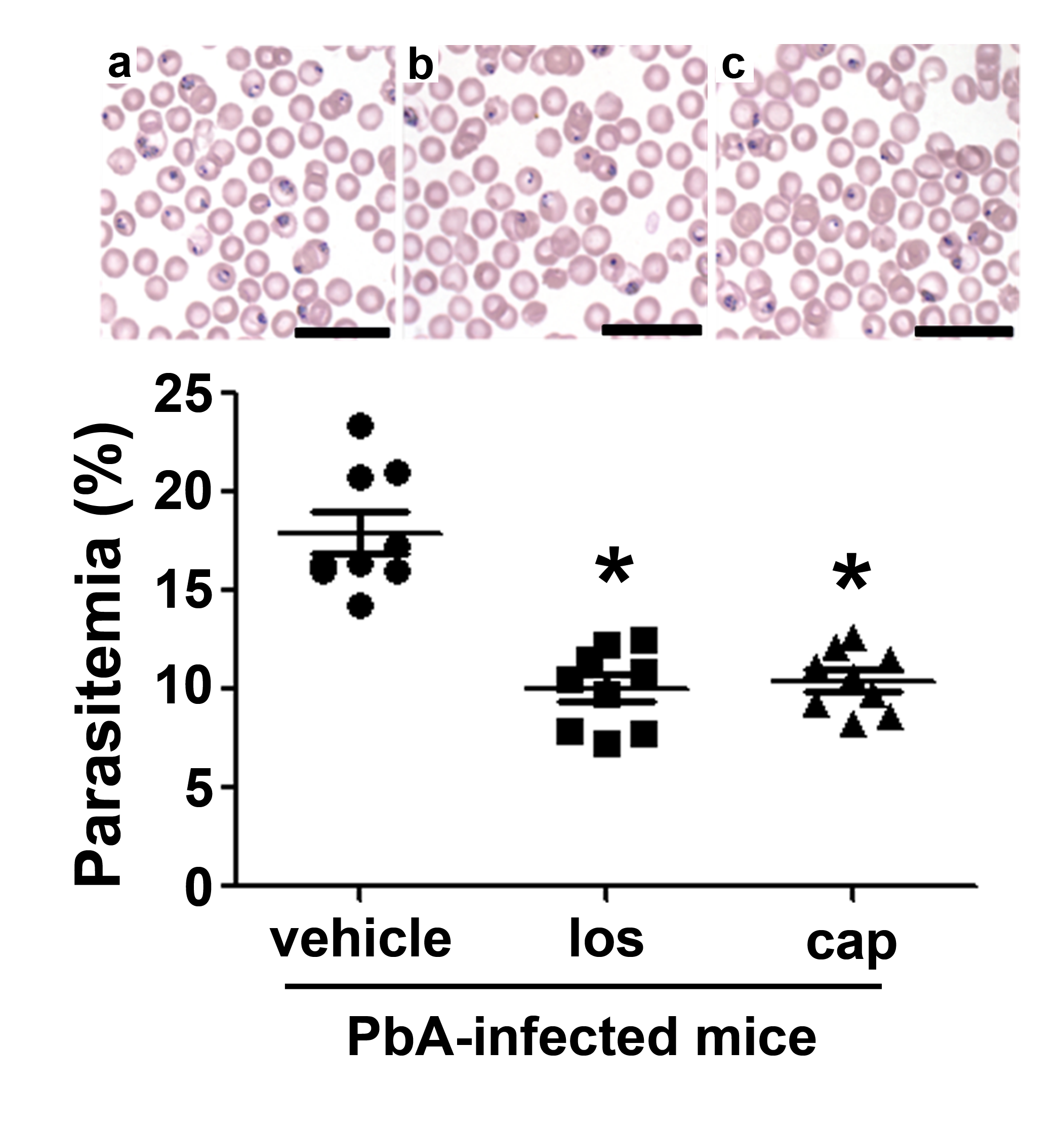

Supplement: S1 Fig — Experimental groups are described in Fig 1 (n = 9 per group). Peripheral blood parasitemia was determined in a blood smear stained with Diff-Quick. Scale bar, 20 μm. The results are expressed as means ± SE. Statistically significant in relation to vehicle (*P < 0.05). (TIF) [file pone.0203836.s001.tif]
